# Local Anesthetic Use in Musculoskeletal Injections

**DOI:** 10.31486/toj.22.0061

**Published:** 2022

**Authors:** Meghan Caballero, Yuka Kobayashi, Andrew W. Gottschalk

**Affiliations:** ^1^ Department of Orthopaedic Surgery, Medical College of Wisconsin, Milwaukee, WI; ^2^ Department of Family Medicine and Orthopaedic Surgery, Medical College of Wisconsin, Milwaukee, WI; ^3^ Department of Orthopedics, Sports Medicine Institute, Ochsner Clinic Foundation, New Orleans, LA

## CASE PRESENTATION

An obese and otherwise healthy 62-year-old male presents to the clinic with chronic but worsening right medial knee pain. Pain is worse with weight-bearing, especially when descending hills and stairs, and disrupts his sleep. Physical examination reveals a trace effusion, tenderness to palpation over the medial joint line, and mild crepitus appreciated with range of motion. Rosenberg/lateral/sunrise radiographs reveal medial joint space narrowing and small medial and patellofemoral compartment osteophytes, consistent with osteoarthritis. He is counseled on weight loss, prescribed a course of physical therapy, and encouraged to use nonsteroidal anti-inflammatory drugs as needed. He is also offered a joint injection for more immediate relief. He asks if the numbing medications used in joint injections are safe.

## BACKGROUND

Injection of local anesthetics with or without corticosteroids is used for diagnosis and treatment of pain associated with a variety of musculoskeletal conditions. Local anesthetics work by binding to and inhibiting voltage-gated sodium channels on nerve cell membranes, thereby preventing development of an action potential and blocking nerve transmission.^1^ They preferentially affect Aγ spindle efferents and Aδ nociceptive fibers in a concentration-dependent manner while relatively sparing unmyelinated C fibers.^1^

Local anesthetics are divided into 2 main categories: esters and amides. Lidocaine, bupivacaine, and ropivacaine are examples of amides and are the most commonly used local anesthetics in peripheral joint injections.^2^ Local anesthetics can be distinguished based on various chemical properties, including lipid solubility, protein binding, and acid dissociation constant (pKa), which impact potency, duration of action, and onset of action, respectively.^3^ The more lipid-soluble an anesthetic is, the more easily it is able to affect the nerve membrane, thus increasing its potency. Local anesthetics with higher protein binding have a longer duration of action and lower bioavailability. Onset of action depends on the proportion of nonionized drug in the solution, which, for bases, is inversely related to the pKa; therefore, local anesthetics with low pKa have a faster onset of action and vice versa. Inflamed tissue is more acidic than healthy tissue and can reduce both the protein binding and the amount of nonionized drug available to provide analgesia.^1^

Awareness of side effects is also important when using local anesthetics. The most serious side effects from local anesthetics are cardiac and central nervous system (CNS) toxicity, specifically if injected intravascularly or intrathecally.^4^ Because the nervous system is more sensitive to local anesthetics than the cardiovascular system, CNS toxicity—consisting of “shivering, muscle twitching, tremor, hypoventilation, respiratory arrest, and…convulsions”—will be detected first.^3^ Systemic toxicity is associated with several risk factors, including drug selection, patient population, and procedure.^5^ To reduce the risk of toxicity, the provider should use the minimum dose of local anesthetic that can provide the desired analgesia without exceeding the maximum dose.

Recent (2009-2019) research has demonstrated toxicity of local anesthetics to human chondrocytes and tenocytes.^6-12^ Local anesthetic toxicity is dependent upon drug type, concentration, use of adjuvant medication such as steroids or epinephrine, and underlying tissue pathology.^2,3,6-12^ Many studies, however, focus on a simulation of high volumes of local anesthetics (30 to 60 cc) as would be used during an arthroscopic procedure, as opposed to a single intra-articular injection.^3^ In this review, we explore the properties of individual local anesthetics for intra-articular and peritendinous injections to help guide the practitioner in making a safe and effective choice.

## REVIEW OF EVIDENCE

The potency of the 3 local anesthetics is directly related to lipid solubility, which is lowest in lidocaine and highest in bupivacaine.^3^ Lidocaine has the lowest pKa and protein solubility and therefore the fastest onset of action (<2 minutes) and shortest duration of action (30 to 120 minutes).^1,3,13^ In contrast, bupivacaine has the slowest onset of action (2 to 10 minutes) and longest duration of action (180 to 360 minutes).^1,3^ Ropivacaine has a similar onset of action to bupivacaine, given their similar pKa values, although its duration of action generally falls between that of lidocaine and bupivacaine (140 to 200 minutes).^3,14^

With regard to side effects, the propensity for CNS toxicity is lowest with lidocaine and highest with bupivacaine based on animal models and appears to be related to lipophilicity and potency of the medication.^15,16^ Both lidocaine and bupivacaine bind to and block cardiac sodium channels; however, bupivacaine does so with a higher affinity and slower dissociation, thus resulting in higher risk of adverse cardiac effects.^1,17^ Ropivacaine, a levo enantiomer of bupivacaine, was created in part because of this cardiac toxicity, as it has lower potency at myocardial sodium and potassium channels.^1^ Ropivacaine also has more vasoconstrictive properties compared to lidocaine and bupivacaine, which can prolong duration of action and delay systemic absorption.^5^ Additionally, compared to lidocaine and bupivacaine, ropivacaine has a higher cardiovascular collapse to CNS ratio, which is “the ratio of drug dose required to cause catastrophic cardiovascular collapse to the drug dose required to produce seizures,”^17^ meaning that CNS features will be detected earlier and allow for treatment of systemic toxicity prior to cardiovascular compromise.^18^

The effect on chondrocytes should also be considered when choosing local anesthetics. Local anesthetics are chondrotoxic via apoptosis, necrosis, mitochondrial dysfunction, extracellular matrix damage, decreased DNA-normalized glycosaminoglycan expression, caspase inhibition, and decreased cell viability.^6,19-23^ A systematic review by Jayaram et al summarized 16 articles published between 2008 and 2018 and reported that ropivacaine has the least chondrotoxicity at doses ≤0.5%, while bupivacaine has the most chondrotoxicity at doses ≥0.5%.^6^ Effects were dose- and time-dependent and worsened by the addition of corticosteroids. All of the studies examined human cartilage after exposure to local anesthetics for set time points ranging from 15 minutes to 24 hours, and the majority compared multiple local anesthetics.^6^ However, all chondrocytes were studied in vitro, making it difficult to conclude the true effect on in vivo cartilage as the exact duration of exposure to local anesthetics following a single intra-articular injection is unclear.^6^ A more recent 2019 study done in vitro using human knee chondrocytes demonstrated similar findings to Jayaram et al, with ropivacaine having the lowest potential for chondrotoxicity.^2^

The toxic effect of local anesthetics on tenocytes has also been studied. Scherb et al demonstrated that in vitro exposure of human tenocytes to bupivacaine resulted in detrimental effects to both proliferation potential and collagen production compared to phosphate-buffered saline.^11^ Piper et al compared the effects of exposure of cultured bovine tenocytes to normal saline, 1% lidocaine, 2% lidocaine, 0.2% ropivacaine, 0.5% ropivacaine, dexamethasone, and combinations of local anesthetic and steroid.^9^ They found that a 30-minute exposure to lidocaine alone resulted in a significant, dose-dependent toxicity, whereas exposure to ropivacaine did not result in similar findings.^9^ When dexamethasone was added to local anesthetics, it potentiated tenocyte toxicity of 0.5% ropivacaine but not either dose of lidocaine.^9^

An additional study by Nuelle et al echoed the above findings and found significant negative effects in harvested canine tenocyte viability and metabolism following 1 and 7 days of exposure to 1% lidocaine as well as after 7 days of exposure to 0.25% bupivacaine.^12^ Of note, Nuelle et al found no significant differences between saline control and 1 day of exposure to 0.25% bupivacaine.^12^ Sung et al compared the effects of various concentrations of lidocaine, bupivacaine, and ropivacaine on cultured human rotator cuff tenofibroblasts and reported that overall toxicity was dependent on exposure time and concentration, with 0.2% ropivacaine being least toxic and lidocaine being very toxic even at lower concentrations.^10^

Finally, Busse et al in 2019 compared cell viability of both human knee chondrocytes and biceps brachii tenocytes following intra-articular injection of lidocaine hydrocholoride 1%, bupivacaine 0.5%, triamcinolone acetonide, dexamethasone 21-palmitate, tranexamic acid, iodine contrast media, hyaluronic acid, and distilled water.^7^ The study was performed in vitro and demonstrated strong toxic effects of local anesthetics and triamcinolone acetonide on both chondrocytes and tenocytes in low dilutions (1:2).^7^

## TAKEAWAY

Local anesthetics are important diagnostic and treatment tools for multiple musculoskeletal pathologies. While lidocaine, bupivacaine, and ropivacaine are the most commonly selected local anesthetics for peripheral joint injections, the choice of specific local anesthetic is largely dependent on the procedure type, dose and concentration, cost, and patient and provider preference. Bupivacaine has the highest potency and provides the longest duration of action, although it has been shown to have an increased risk of adverse effects, including cardiac, CNS, and chondrocyte toxicity.^1,17^ Lidocaine has been shown to have a high potential for tenocyte toxicity even at low concentrations.^6,9,10^ Ropivacaine has generally been found to be least toxic to chondrocytes and tenocytes among the 3 local anesthetics and therefore may be a more desirable choice.^2,6,10^ However, ropivacaine costs significantly more than both lidocaine and bupivacaine, making it a difficult option for some providers.

The Table presents a comparison of lidocaine, bupivacaine, and ropivacaine with regard to their use in musculoskeletal joint and tendon injections.^1-3,5-10,12-18,23-27^

**Table.** Comparison of Commonly Used Local Anesthetics for Musculoskeletal Injections

Characteristic	1% Lidocaine Hydrochloride	2% Lidocaine Hydrochloride	0.25% Bupivacaine Hydrochloride	0.5% Bupivacaine Hydrochloride	0.5% Ropivacaine Hydrochloride
Trade name(s)	Xylocaine		Marcaine, Sensorcaine		Naropin
Lipid solubility^3^	25		346		115
Potency^1^	+		+++		++
pKa^1,3^	7.8		8.1		8.1
Onset of action, minutes^1,3,13,14^	Fast (<2)		Long (2-10)		Moderate (<2-10)
Protein binding, %^1^	70		95		94
Duration of action, minutes^3,23^	30-120		180-360		140-200
Cardiac toxicity^1,3,5,17,18^	++		+++		+
Central nervous system toxicity^3,5,15,16^	+		+++		++
Chondrocyte toxicity^2,3,6-8^	++		++		+
Tenocyte toxicity	Potential for toxicity that appears to be greater than other local anesthetics^9,10,12^		Potential for toxicity^7,11,12^		Potential for toxicity when combined with dexamethasone^9^
Typical dose, mL^3,24-26^	3-5		0.5-2		2-4
Maximum dose without epinephrine, mg/kg^1,3,5,27^	3-5		2		3
Cost	+		++		+++

Note: + signifies least and +++ signifies most.

pKa, acid dissociation constant.

## CASE RESOLUTION

The patient underwent a right knee injection using a nonguided inferolateral approach using a combination of 2 mL of 0.5% ropivacaine and 1 mL of 40 mg/mL triamcinolone acetonide. He was compliant with his physical therapy and home exercise regimen following the injection and reported 5 months of significant improvement in his pain. The patient was not interested in surgical intervention, and multiple injections may need to be considered in his treatment course during the next several years. Therefore, ropivacaine was the agent of choice because of its relative low risk of adverse effects and chondrocyte toxicity.

## Notes

*This article meets the Accreditation Council for Graduate Medical Education and the American Board of Medical Specialties Maintenance of Certification competencies for Patient Care and Medical Knowledge.*
